# Characteristic odour in the blood reveals ovarian carcinoma

**DOI:** 10.1186/1471-2407-10-643

**Published:** 2010-11-24

**Authors:** György Horvath, Håkan Andersson, Gunnar Paulsson

**Affiliations:** 1 Department of Oncology, Sahlgrenska University Hospital, Göteborg, Sweden; 2 Department of Obstetrics and Gynaecology, Skövde City Hospital, Skövde, Sweden

## Abstract

**Background:**

Ovarian carcinoma represents about 4% of all cancers diagnosed in women worldwide. Mortality rate is high, over 50%, mainly due to late diagnosis. Currently there are no acceptable screening techniques available, although ovarian cancer belongs to the group of malignancies for which mortality could be dramatically reduced by early diagnosis.

In a recently published study, we clearly demonstrated that human ovarian carcinoma tissues can be characterized by a specific odour, detectable by a trained dog. Another recent study confirmed these results using an electronic nose.

**Methods:**

In the present work, we examined whether the cancer-specific odour can also be found in the blood. Two specially trained dogs were used. Both ovarian cancer tissues and blood from patients with ovarian carcinoma were tested.

**Results:**

The tissue tests showed sensitivity of 100% and specificity of 95%, while the blood tests showed sensitivity of 100% and specificity of 98%.

**Conclusions:**

The present study strongly suggests that the characteristic odour emitted by ovarian cancer samples is also present in blood (plasma) taken from patients with the disease. This finding opens possibilities for future screening of healthy populations for early diagnosis of ovarian carcinoma. A future challenge is to develop a sensitive electronic nose for screening of ovarian carcinoma by testing the blood/plasma to detect the disease at a stage early enough for treatment to be effective.

## Background

Worldwide, there are more than 204,000 new cases of ovarian cancer annually, accounting for around 4% of all cancers diagnosed in women. Incidence rates vary considerably, with the highest rates in the United States and Northern Europe and the lowest rates in Africa and Asia. Around 43,000 cases occur each year in Europe, and 22,000 in the USA. In Sweden, the disease represents 3.1% of all cancer cases in women, totalling about 900 cases per year. Despite this relatively low incidence rate, it is the fifth most common cause of cancer death in women.

Because of the high mortality rates, ovarian cancer is one of several diseases that fulfil some of the criteria necessary for the introduction of population screening: it is an important health problem, and early detection is associated with improved outcomes. Potential screening tests for ovarian cancer have not yet been shown to reduce mortality, although both ultrasound and tumour markers can detect a significant proportion of ovarian cancers preclinically. Currently, there is no accepted screening programme for ovarian cancer [1-3].

In a recently published study, we clearly demonstrated that human ovarian carcinoma tissues can be characterized by a specific odour, detectable by a trained dog. The same study showed that a dog can be trained to distinguish between different histopathological types and grades of ovarian carcinomas, including borderline tumours, as well as different healthy control samples[4]. Double-blind tests showed 100% sensitivity and 97.5% specificity. Moreover, the odour of ovarian carcinomas seems to differ from those of other gynaecological malignancies, such as cervical, endometrial, and vulvar carcinomas, suggesting that different malignancies have different odour characteristics. The study further showed that early-stage and low-grade ovarian carcinomas emit the same specific smell as advanced tumours. These results suggest that the specific cancer odour may be used for screening, early diagnosis, and differential diagnosis of different malignant diseases in the future, when it becomes technologically possible.

Detection of other malignancies by dogs, such as melanoma [5] and bladder[6], breast, and lung cancer[7], has also been reported in peer-reviewed scientific journals.

Besides dog studies, different technical methods such as gas chromatography and mass spectrometry (GC/MS),[8] gas chromatography (GC)-based arrays,[9] and nanoparticle-polymer sensor arrays[10] have been used to detect malignant cells in vitro.

In our last study [11], volatile signals emitted by human seropapillary ovarian carcinoma samples and healthy tissues such as fallopian tube, myometrium, and postmenopausal ovarium were analyzed using an electronic nose. The electronic nose correctly classified 84.4% of cancerous tissues and 86.8% of the control material. These results confirm the basic results from our dog study; that is, the ovarian cancer samples emit specific odour/volatile signals. Although the study was small, the results offer some indication that early electronic detection of ovarian carcinoma may be possible.

One important challenge in this line of cancer research is to find suitable target(s) for diagnostic use; the blood offers a possible option.

The aim of this study was to test whether the specific odour emitted by ovarian carcinomas and borderline ovarian tumours can be detected by trained dogs in blood from patients with these diseases.

## Methods

### The dogs

Two dogs were used: Hanna, a 7-year-old black Giant Schnauzer (chip no. 967000000389928), and Lotti, a 3-year-old black Giant Schnauzer (chip no. 098100311386). Hanna was previously trained to detect ovarian carcinoma samples, and the test results were published in 2008[4]. In the present study, she was trained over the course of 9 months to detect blood samples from ovarian carcinoma patients, and during this time she did not sniff carcinoma samples. Lotti, who had not previously been trained, was trained during the same time period to detect ovarian carcinoma samples. Lotti had never sniffed blood samples before the test series.

### Training

The training method is described in detail elsewhere;[4] a brief overview is given below.

#### Learning odour signature

Training was initially designed as a selection model, resembling the training of sniffer dogs. In brief, the dog was encouraged to sniff a few rags attached to pieces of string and placed on the floor. One of them contained an ovarian cancer sample. When the dog showed interest in the target, the handler quickly snatched it away. This was repeated several times.

#### Learning odour discrimination

When the dog was capable of identifying even low concentrations of the target vapour (finding the hidden tumour), we began using non-target odours as controls. Target and non-target samples were placed in glass containers, which were covered with perforated lids and placed inside wooden boxes (25 cm × 25 cm × 25 cm). The boxes and containers were cleaned with 95% alcohol after each run. We initially used only one control specimen, and the dog was permitted to choose the right parcel and disregard the control. Step by step, we increased the number of control samples to five (the combination of five controls and one target was considered a run). To minimize external influence, the exercises were carried out in several training rooms in random sequence.

#### Learning to distinguish extraneous odours

Although target and control samples were handled carefully in this phase of the training, other components such as boxes and glass containers were contaminated by different individuals, including the handler. However, this contamination had no observable influence on the dog's target identification during this last period of training.

### Tumour and blood samples

Ovarian carcinoma samples consisting of different histopathological types of various grades and stages, including borderline tumours, were used during the training period[4]. Tumour material was collected at primary surgery, before chemotherapy. It was taken from the primary tumour in the pelvis (from the ovarium if possible, at early stages) but not from the peritoneum. All tumours were assessed by the same pathologist in accordance with regional treatment guidelines for gynaecological malignancies in western Sweden. The tumour samples were stored in small plastic tubes, preserved immediately at -20°C, and transported to our tumour bank (Ethical Committee license number: S-154-02), where they were kept at -80°C. Each tumour was divided into 10-30 samples of about 3 mm × 3 mm × 3 mm, and thawed at room temperature for 15-30 minutes before being used in the training. For the test sections, other gynaecological tumour samples, such as cervical, vulvar, and endometrial carcinomas, were also taken from this tumour bank and treated identically to the ovarian carcinomas. Sample imprints for cytological examination were performed on all tumours and all controls. The imprints were examined to verify the presence of malignancy (established when at least 75% of cells were malignant). Controls were accepted only in the complete absence of malignant cells.

Blood samples were obtained before primary surgery from patients with ovarian carcinoma and from patients with cervical, vulvar, and endometrial carcinomas. These samples were taken in EDTA tubes, then centrifuged at 3000 rpm for 10 min. and plasma pots over the small plastic tubes. The rest of the plasma samples after undergoing CA-125 analysis, were kept at -80°C in our tumour bank (Ethical Committee license number: S-220-08). Blood samples with >500 IU CA-125 values were used for training, with one drop being placed in a small plastic dish inside each box.

Median donor age was 67 years (range: 35-79) for tissue samples and 63 years (range: 45-77) for blood samples. Tissue and blood samples used during the training period were not used in the tests.

### Controls

Abdominal fat and muscle (myomas), and healthy postmenopausal ovarium samples were used as controls. Control blood (plasma) samples were collected from young, healthy female individuals. However, in some cases we also used blood samples from male individuals, including handlers. This had no observable influence on the dog's target identification. Control plasma samples were treated identically to the targets. Median donor age was 65 years (range: 40-81) for tissue samples and 41 years (range: 27-67) for blood samples. Tissues and blood samples used during the training period were not used in the tests.

### Test design

Tests were carried out according to the double-blind principle; both test leader and handler were blinded to the location of the target samples, and were present in the test location only when the dogs were working. The dogs were tested in four sections, two on day 1 and two on day 2. Each section was composed of ten runs, and each run included six boxes; five of the boxes contained control materials and the remaining box contained the target material. Placement of the target box was changed by an outside assistant between each run. Section 1 (day 1): Lotti sniffed tissues; Section 2 (day 1): Hanna sniffed blood; Section 3 (day 2): Lotti sniffed blood; Section 4 (day 2): Hanna sniffed tissues. The tests were documented on paper and DVD.

### Dog's response

A positive response was defined as indicating the target box by scratching with foreleg(s) and lying down or sniffing at, but not indicating the control samples. A negative response was defined as indicating a control box and not indicating the target.

### Statistical methods

Sensitivity and specificity were calculated in the same way as for diagnostic testing. That is, the sensitivity (or the true positive rate) of the test was the proportion of cancer samples that were correctly identified by the dog, and the specificity (or the true negative rate) was the proportion of control samples negatively indicated by the dog.

Binomial probability distribution was used to compare the performance of the dog with a random selection of sample boxes. Each test consisted of ten runs, each of which included one target sample and five controls. Under the assumption of random positive indication by the dog, the number of correctly identified runs was a binomial distribution with a 1/6 probability of success (Table 1).

**Table 1:** Calculated sensitivity and specificity


**Dog indication**	**Cancer**	**Control**	**Sample**

positive	a	b	a + b

Negative	c	d	c + d

	a + c	b + d	n

Sensitivity = a/(a + c), and specificity = d/(b + d).

## Results and Discussion

Section 1: The dog correctly identified all cancer samples, giving a sensitivity of 100%. Two controls out of 50 were indicated, giving a specificity of 96% (Tables 2 and 3).

**Table 2:** Dog's responses in Section 1 (Dog: Lotti; Material: tissues)

Box 1	2	3	4	5	6
Corp58	Myoma	Myoma	**1786**	Myoma	V

Corp58	Myoma	Fat	Myoma	**285**	V

Corp58	**443**	Myoma	Myoma	Ov	V

Corp58	Myoma	**5377**	Myoma	Myoma	Fat

**425**	Fat	Corp58	Myoma	Myoma	V

Myoma	Myoma	Coll2	**270**	Fat	Myoma

Myoma	Ov	Corp73	V	Myoma	**5005**

Fat	**5011**	Corp58	Fat	Myoma	Myoma

Myoma	Myoma	Corp73	Fat	**5039**	Ov

Myoma	Fat	**466**	V	Fat	Myoma

**xxxx **= target

Corp = endometrial carcionoma; Coll = Cervical carcinoma; V = vulvar carcinoma; Myoma = muscle from uterine walls (healthy individuals); Fat = abdominal, intraperitoneal fats from healthy individuals; Ov = healthy postmenopausal ovarium samples

Dog's responses:

Positive (**bold**)

Negative (monospace)

**Table 3:** Clinicopathological features in Section 1 (Dog: Lotti; Material:tissues)

Tissue	CA-125 U/ml	Histology	Grade	Stage	Diagnosis
1786	> 200	seropapillary	2	III/C	Ca. ovari

285	< 35	mucinous		III	Borderline

443	500	seropapillary	3	III/C	Ca. ovari

5377	100	mucinous	1	I/A	Ca. ovari

425	154	serous		III/A	Borderline

270	180	carcinosarcoma		III/B	Ca. ovari

5005	< 35	endometroid	1	I/B	Ca. ovari

5011	80	endometroid	3	II/B	Ca. ovari

5039	195	carcinosarcoma		II/B	Ca. ovari

466	-	mucinous	3	III/B	Ca. ovari

V	< 35	squamous	2	II	Ca. vulvae

Corp58	< 35	endometroid	3	II/A	Ca. corp. ut.

Corp73	< 35	endometroid	2	I/B	Ca. corp. ut.

Coll2	< 35	squamous	3	III/A	Ca. colli ut.

The probability of the dog getting at least 8 out of 10 runs completely correct entirely by chance (assuming the two indicated controls belonged to different runs) was 8.43*10^-7^.

Section 2: The dog correctly identified all plasma samples, both from cancer patients and healthy controls, giving sensitivity and specificity of 100% (Tables 4 and 5).

**Table 4:** Dog's responses in Section 2 (Dog: Hanna; Material: plasma)

Box 1	2	3	4	5	6
V	**8783**	Corp1	XX	Coll	XX

V	X	Corp1	XX	Coll	**3622**

V	XX	X	**1200**	Corp1	XX

**3712**	X	Corp2	V	Coll	XX

Coll	XX	Corp1	V	**3607**	X

Coll	XX	**2246**	V	Corp1	Corp2

**3609**	X	Coll	V	Corp2	XX

XX	XX	Coll	V	Corp2	**2124**

X	**2192**	Coll	XX	Corp2	XX

XX	Corp2	Coll	V	**3654**	XX

**xxxx**= target

V = vulvar carcinoma; Coll = cervical carcinoma; Corp = endometrial carcinoma; × = plasma obtained from healthy female individuals; XX = plasma obtained from healthy male individuals

Responses:

Positive  (**bold**)

Negative  (monospace)

**Table 5:** Clinicopathological features in Section 2 (Dog: Hanna; Material: plasma)

Plasma	CA-125	Histology	Grade	Stage	Diagnosis
8783	< 35	mucinous		I/A	Borderline

3622	< 35	endometroid	3	III/B	Ca. ovari

1200	> 500	seropapillary	2	III/B	Ca. ovari

3712	< 35	mucinous		II/B	Borderline

3607	< 35	seropapillary	3	III/C	Ca. ovari

2246	> 100	seropapillary	2	III/C	Ca. ovari

3609	< 35	adenocarcinoma	2	I/B	Ca. ovari

2124	> 500	seropapillary	3	III/C	Ca. ovari

2192	> 500	seropapillary	2	III/A	Ca. ovari

3654	< 35	adenocarcinom	3	IV	Ca. ovari

Vulva	< 35	squamous	2	II	Ca. vulvae

Corp 1	< 35	endometroid	3	I/C	Ca. corp. ut.

Corp 2	< 35	endometroid	3	I/C	Ca. corp. ut.

Coll	< 35	adenocarcinoma	2	II/A	Ca. colli ut.

The probability of the dog getting all 10 runs completely correct entirely by chance was 1.65*10^-8^.

Section 3: The dog correctly identified all plasma samples taken from patients with ovarian carcinoma (sensitivity = 100%), and also indicated two out of 50 control samples, giving a specificity of 96% (Tables 6 and 7).

**Table 6:** Dog's responses in Section 3 (Dog: Lotti; Material: plasma)

Box 1	2	3	4	5	6
V	X	Corp1	X	Coll	**2124**

X	XX	**3646**	X	Corp1	Coll

**7673**	XX	V	X	Coll	Corp1

Coll	X	V	XX	**2192**	Corp1

Coll	**6647**	X	XX	X	Corp1

Coll	Corp2	V	X	XX	**3631**

X	Corp2	V	**2139**	X	X

**3657**	Corp1	X	Coll	XX	XX

XX	X	Corp1	Coll	**2144**	X

XX	Corp2	**3635**	Coll	V	X

**xxxx **= target

V = vulvar carvinoma; Coll = cervical carcinoma; Corp = endometrial carcinoma; × = plasma

obtained from healthy female individuals; XX = plasma, obtained from healthy male individuals

Responses:

Positive (**bold**)

Negative (monospace)

**Table 7:** Clinicopathological features in Section 3 (Dog: Lotti; Material: plasma)

Plasma	CA-125	Histology	Grade	Stage
2124	> 500	seropapillary	3	III/C

3646	< 35	endometroid	2	III/B

7673	< 35	mucinous	--	I/C

2192	> 500	seropapillary	2	III/A

6647	< 35	mucinous	-	I/B

3631	< 35	seropapillary	2	III/B

2139	> 200	carcinosarcom	-	III/A

3657	< 35	seropapillary	1	III/C

2144	> 500	adenocarcinoma	3	II/A

3635	< 35	seropapillary	3	III/B

Vulva	< 35	squamous	2	II

Corp 1	< 35	endometroid	3	I/C

Corp 2	< 35	endometroid	3	I/C

Coll	< 35	adenocarcinoma	2	II/A

The probability of the dog getting at least 8 out of 10 runs completely correct entirely by chance (assuming the two indicated controls belonged to different runs) was 8.43*10^-7^.

Section 4: The sensitivity was again 100%, and the dog indicated 3 out of 50 control tissues including other gynaecological carcinomas, giving a specificity of 94% (Tables 8 and 9).

**Table 8:** Dog's responses in Section 4 (Dog: Hanna; Material: tissues)

Box 1	2	3	4	5	6
Fat	**258**	Myoma	V	Corp	V

Corp58	Myoma	Fat	Myoma	Corp	**1786**

**5005**	Fat	Myoma	Myoma	Ov	V

Corp58	Myoma	Fat	**5039**	Myoma	Fat

V	Fat	Fat	Fat	Corp	**147**

Fat	V	**425**	V	Fat	Fat

Myoma	**5011**	Fat	Fat	Corp73	Fat

V	Corp58	Corp58	Fat	**270**	Myoma

**5377**	Myoma	Corp73	Fat	V	Ov

Myoma	Fat	Fat	**443**	Fat	Myoma

xxxx = target

Corp = endometrial carcinoma; Coll = Cervical carcinoma; V = Vulvar carcinoma; Myoma = muscle from uterine walls (healthy individuals); Fat = abdominal, intraperitoneal fats from healthy individuals; Ov = healthy postmenopausal ovarium samples

Dog's responses:

Positive (**bold**)

Negative (monospace)

**Table 9:** Clinicopathological features in Section 4 (Dog: Hanna; Material: tissues)

Tissue	CA-125 U/ml	Histology	Grade	Stage	Diagnosis
1786	> 200	seropapillary	2	III/C	Ca. ovari

285	< 35	mucinous		III	Borderline

443	500	seropapillary	3	III/C	Ca. ovari

5377	100	mucinous	1	I/A	Ca. ovari

425	154	serous		III/A	Borderline

270	180	carcinosarcoma		III/B	Ca. ovari

5005	< 35	endometroid	1	I/B	Ca. ovari

5011	80	endometroid	3	II/B	Ca. ovari

5039	195	carcinosarcoma		II/B	Ca. ovari

147	-	mucinous	3	II/B	Ca. ovari

V	< 35	squamous	2	II	Ca. vulvae

Corp58	< 35	endometroid	3	II/A	Ca. corp. ut.

Corp73	< 35	endometroid	2	I/B	Ca. corp. ut.

Coll2	< 35	squamous	3	III/A	Ca. colli ut.

The probability of the dog getting at least 7 out of 10 runs completely correct entirely by chance (assuming the three indicated controls belonged to different runs) was 1.94*10^-5^.

When the results were pooled by sample type, the tissue tests showed a sensitivity of 100% and a specificity of 95%, and the plasma tests showed a sensitivity of 100% and a specificity of 98%.

This study is the first presentation of a specific odour emitted by human plasma from ovarian cancer patients. In addition, it reveals the important observation that trained dogs can discriminate between plasma samples from ovarian cancer patients and plasma taken from patients with other malignancies such as endometrial, cervical, and vulvar carcinomas.

The present study also confirms results from our previous work, in which we showed that a trained dog could discriminate different histopathological types and grades of ovarian carcinoma tissues, including borderline tumours, from healthy control samples including postmenopausal ovary. The dog could also discriminate ovarian carcinoma tissues from all other gynaecological malignancies. Sensitivity and specificity rates in the double-blind test series were 100% and 97.5%, respectively[4]. In the present study, the sensitivity and specificity for the two tissue tests (Sections 1 and 4) were 100% 95%, respectively.

The present study strongly suggests that the characteristic odour emitted by ovarian cancer samples is also present in blood (plasma) taken from patients with the disease. This observation suggests that the specific cancer odour in the blood/plasma may be used for screening, diagnosis, and differential diagnosis of different malignant diseases. The past decade has seen an increasing amount of research into different technical methods of identifying the characteristic volatile organic compound (VOC) signals emitted by malignancies. Methods such as gas chromatography and mass spectrometry,[8] gas chromatography-based arrays,[9] and nanoparticle-polymer sensor arrays[10]-"chemical noses" and electronic noses-have been used to detect malignant cells in vitro, and diagnostic methods for lung cancer using exhaled breath have also been investigated[12].

Our recently completed study presents the first evidence that an electronic nose can provide an easy technique to distinguish the VOC signals emitted by human ovarian carcinomas and healthy human fallopian tube, myometrium, and postmenopausal ovary, respectively[11]. The electronic nose showed a sensitivity of 84.4% and a specificity of 86.8% in the total material. However, in that study we did not test our electronic nose on blood/plasma samples or on malignancies other than ovarian carcinoma.

It is not easy to make comparisons between the present study and other dog studies. Two of the available dog studies used training methods that differed from those in the present study, as well as different target materials [6,7]. A third study was, like ours, based on tissue material, but included only a very limited number of tissues [5].

Our observations from dog studies show that trained dogs can detect even as small a quantity as 20 ovarian carcinoma cells on the abdominal fat (data not shown). Thus, the cancer-specific odour/VOC components are emitted even in early phases of tumour development. We believe that a significant challenge for future experiments is to construct more sensitive electronic noses, not only for early detection but also for differential diagnosis between malignancies. If the electronic nose is to be used for screening of ovarian carcinoma by testing the blood/plasma, it must be able to detect the disease in the early stages, when treatment is effective.

It is difficult to compare the sensitivity of a dog's nose to that of the electronic nose. Dogs detect only odour molecules, whereas electronic noses may also detect several odourless compounds (e.g. CH4). The two systems may thus detect different levels of sensitivity. Our experience suggests that trained dogs could be used under controlled circumstances in experiments as complementary "instruments" to further explore this very interesting new property of malignancies. Similar suggestions were published by Gordon et al. [13].

## Conclusion

The present study strongly suggests that the characteristic odour emitted by ovarian cancer samples is also present in blood (plasma) taken from patients with the disease.

## Financial and competing interests

This work was supported by the Folksam Research Foundation, Stockholm; Innovationsbron AB, Göteborg, Sweden; and Royal Canin, Sweden.

The authors had no other relevant affiliations or financial involvement with any organization or entity with a financial interest in or financial conflict with the subject matter or materials discussed in the manuscript, apart from those disclosed.

No writing assistance was used in the production of this manuscript.

The figures and tables presented here are original and have not been presented earlier.

## Authors' contributions

GH: project leader, study design, dog training and handling, coordination of manuscript drafting; HA: collecting blood samples statistical calculations required for the study design, drafting part of the manuscript; GP: collecting tissues (some, which lacking in the tumour bank), drafting part of the manuscript, participating in the study coordination. All authors read and approved the final manuscript.

## Pre-publication history

The pre-publication history for this paper can be accessed here:

http://www.biomedcentral.com/1471-2407/10/643/prepub
